# Beyond the Medial Regions of Prefrontal Cortex in the Regulation of Fear and Anxiety

**DOI:** 10.3389/fnsys.2016.00012

**Published:** 2016-02-22

**Authors:** Yoshiro Shiba, Andrea M. Santangelo, Angela C. Roberts

**Affiliations:** ^1^Department of Physiology, Development and Neuroscience, University of CambridgeCambridge, UK; ^2^Behavioural and Clinical Neuroscience Institute, University of CambridgeCambridge, UK

**Keywords:** anxiety, fear, emotion regulation, prefrontal cortex, orbitofrontal cortex, ventrolateral prefrontal cortex, primate, marmoset

## Abstract

Fear and anxiety are adaptive responses but if left unregulated, or inappropriately regulated, they become biologically and socially maladaptive. Dysregulated emotions are manifest in a wide variety of psychiatric and neurological conditions but the external expression gives little indication of the underlying causes, which are inevitably multi-determined. To go beyond the overt phenotype and begin to understand the causal mechanisms leading to conditions characterized by anxiety and disorders of mood, it is necessary to identify the base psychological processes that have become dysregulated, and map them on to their associated neural substrates. So far, attention has been focused primarily on the medial regions of prefrontal cortex (PFC) and in particular their contribution to the expression and extinction of conditioned fear. However, functional neuroimaging studies have shown that the sphere of influence within the PFC is not restricted to its medial regions, but extends into dorsal, ventrolateral (vlPFC) and orbitofrontal (OFC) regions too; although the causal role of these other areas in the regulation of fear and anxiety remains to be determined and in the case of the OFC, existing findings are conflicting. Here, we review the evidence for the contribution of these other regions in negative emotion regulation in rodents and old world and new world monkeys. We consider a variety of different contexts, including conditioned and innate fear, learned and unlearned anxiety and cost-benefit decision-making, and a range of physiological and behavioral measures of emotion. It is proposed that both the OFC and vlPFC contribute to emotion regulation via their involvement, respectively, in the prediction of future outcomes and higher-order attentional control. The fractionation of these neurocognitive and neurobehavioral systems that regulate fear and anxiety opens up new opportunities for diagnostic stratification and personalized treatment strategies.

## Introduction

Negative emotions such as fear and anxiety are highly adaptive and complex mental states that are the product of interactions between cognition, physiological responses and behaviors. The continuum of emotional responses stretches from unlearnt reflexes and fixed action patterns, through Pavlovian learning (in which novel stimuli, through their association with aversive events, come to elicit conditioned responses, CRs), to instrumental behavior, whereby the organism takes adaptive control of the environment. Via feedback loops between brain and body our fluctuating emotions influence attention, decision making, memory and social interactions. Once the source of fear or anxiety is dealt with or disappears, the emotional responses dissipate so that everyday activity can be resumed. Such effective regulation of negative emotional responses is critical for the physiological, psychological and social well-being of individuals. However, in some, these emotional responses become chronic or exceedingly recurrent even without an apparent external source. Failure to down-regulate or control one’s emotional responses, even when circumstances change from threatening to relatively safe, can have a devastating impact on a sufferer’s life.

Emotional disturbance is a core symptom of mood and anxiety disorders and a prominent symptom of many other neuropsychiatric conditions, including schizophrenia (Braga et al., 2013), obsessive compulsive disorder (Murphy et al., 2013) and autism (van Steensel et al., 2011), being also common in neurodegenerative disorders such as Parkinson’s disease (Dissanayaka et al., 2014) and Huntington’s disease (Dale and van Duijn, 2015). A key feature is clinical anxiety that is categorized by the Diagnostic and Statistical Manual of Mental Disorders (DSM) into different types that are based on differences in symptomatology. The most recent edition (DSM-5) lists seven disorders under the group of Anxiety Disorders (separation anxiety disorder, selective mutism, specific phobia, social phobia, panic disorder, agoraphobia and generalized anxiety disorder, GAD). Closely related but separate groups from the Anxiety Disorders are obsessive-compulsive disorder (OCD) and posttraumatic stress disorder (PTSD). As indicated by this heterogeneity at the level of symptomatology and disorders, anxiety is a multifaceted phenomenon physiologically, behaviorally and cognitively.

Despite the continuous effort within the clinical communities to refine the diagnostics and improve treatments, considerable challenges remain, one of which is marked individual variation in the response to treatments. For example, whilst selective serotonin reuptake inhibitors (SSRIs) are considered as the first-line pharmacological treatment for anxiety disorders and depression, up to 40% of patients are estimated to be partially or completely resistant to the treatment (Bystritsky, 2006; Ipser et al., 2006). Thus, two patients diagnosed with the same anxiety disorder with apparently similar symptoms may respond differently to the same treatment suggesting that although the outward observable symptoms appear similar, the underlying neurobiological mechanisms may differ. Conversely, there are also extensive comorbidities between different anxiety disorders as well as between an anxiety disorder and depression. This increases the difficulty of selecting a specific treatment (Bystritsky et al., 2013) and points to the possibility that either a common neural network contributes to distinct psychiatric conditions or the networks are distinct but equally vulnerable to external or internal stressors. Whilst these appear mutually exclusive, a recent neuroimaging study of anxious temperament in macaques reported that variation in the expression of three different dimensions of the anxious phenotype (cortisol, freezing and vocalization responses to a potential threat) was predicted by metabolic change in three different brain regions (lateral anterior hippocampus, motor cortex and vlPFC, respectively) but there was also common neural substrates (central nucleus of the amygdala and anterior hippocampus) that were shared by all three dimensions (Shackman et al., 2013). These findings reinforce the proposal that improvements in treatments require more refined and biologically-valid diagnostic approaches, which require stratification of mood and anxiety disorders based on the underlying neurobiological mechanisms (Insel et al., 2010).

Much of our knowledge of the neurobiological mechanisms underlying negative emotion has come from studies of Pavlovian fear conditioning in rodents, which have revealed the basic neural circuitry of the acquisition and expression of defense responses. In Pavlovian fear conditioning, an initially neutral cue acquires affective properties (conditioned stimulus, CS) through repeated temporal pairings with an aversive event such as a foot shock (unconditioned stimulus, US). Sensory information of both CS and US are transmitted from peripheries to respective sensory processing areas and subsequently converge at the amygdala where the association of the two stimuli occurs. Through its connections to the brainstem, hypothalamus and motor areas, the amygdala is critically involved in the physiological and behavioral response to a learnt (i.e., conditioned) or innate threat, especially when the threat is imminent and explicit (LeDoux, 2007; Walker et al., 2009). The connection between the amygdala and the bed nucleus of stria terminalis (BNST) is involved in a longer-lasting state of apprehension to a sustained and diffuse threat (Davis et al., 2010). A projection from the hippocampus to the amygdala has been shown to be critical for contextual association of fear experience (LeDoux, 2000). Amygdala projections to the sensory cortices and inferior temporal cortex influence automatic selective attention to a potential threat (Bishop, 2007; Duncan and Barrett, 2007). However, we are only beginning to gain insight into the neural mechanisms underlying the regulation of this circuitry, emanating primarily from the prefrontal cortex (PFC) and anterior cingulate cortex (ACC).

**Figure 1:**
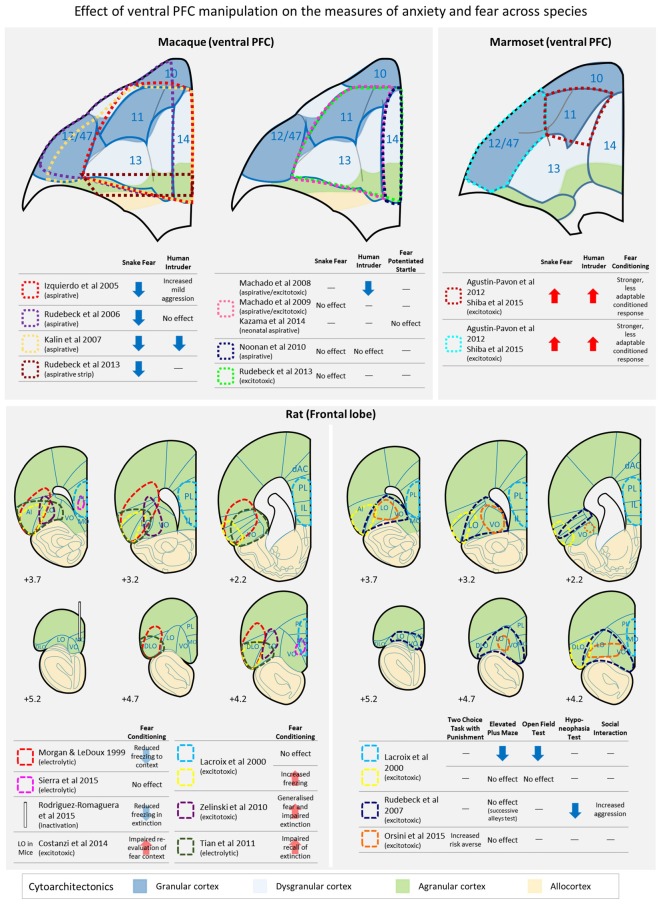
**A schematic diagram depicting the location of lesions across studies in primates (upper panel) and rats (lower panel) in which the effects of ventral prefrontal cortex (PFC) damage on a variety of anxiety and conditioned/unconditioned fear tests have been investigated.** AI, anterior insula; dAC, dorsal anterior cingulate; DLO, dorsolateral orbital area; IL, infralimbic cortex; LO, lateral orbital area; MO, medial orbital area; PL, prelimbic cortex; VO, ventral orbital area. Cytoarchitectonics of the ventral PFC are based on Carmichael and Price (1994; rhesus macaques), Burman and Rosa (2009; marmosets), Paxinos and Watson (2006; rats) and cortical granularity taken from Wise (2008).

The human PFC is located in the anterior portion of the frontal lobe and broadly includes cytoarchitectonically defined Brodmann areas (BA) 8, 9, 10, 11, 13, 14, 44, 45, 46 and 47/12 (Petrides and Pandya, 1994; Öngür et al., 2003). It contains three types of cortices, granular (BA 8, 9, 10, 11 and 12), dysgranular (BA 13 and 14) and agranular (posterior BA 13 and 14, and Insula) based on the existence or not of a layer 4, which is well populated with tiny granule cells and receives a major afferent projection from the mediodorsal nucleus of the thalamus. The agranular cortices are considered as transition areas from an evolutionally older allocortex to the granular cortex (Wise, 2008). Whilst primates, including humans possess all three types of cortices in the frontal region, rodents and other mammals lack the granular and dysgranular cortices (Preuss, 1995; see Figure 1). Human ACC lies at the midline, forming a collar around the genu of the corpus callosum and includes the cingulate gyrus and sulcus, comprising BA 24, 25 and 32 (Vogt and Paxinos, 2014). In studies of neuroimaging, neurophysiology and behavioral neuroscience, these prefrontal and cingulate areas are often grouped into larger dorsal and ventral regions. The dorsal region includes the dorsolateral PFC (dlPFC), which broadly spans BA 8, 9 and 46. The ventral region is typically subdivided into the ventromedial PFC (vmPFC; posterior part of BA 10 and 14, as well as subgenual (sg) and perigenual anterior cingulate areas, 25, 32), vlPFC (BA 47/12 and 45) and orbitofrontal (OFC; primarily BA 11 and 13).

An understanding of the role of the PFC and ACC in regulating subcortical emotion circuitry has come from two major sources. Experimental studies in rodents have focused primarily on the ventral regions of ACC and their contribution to the extinction of conditioned fear, through modulation of amygdala activity. The ventral regions of ACC are often referred to in the literature as vmPFC or medial PFC, which includes the prelimbic (PLc) and infralimbic (ILc) cortices (Krettek and Price, 1977; but see Vogt and Paxinos, 2014 which subdivides this region into areas 25 and 32). Extinction of a conditioned fear response occurs as a consequence of repeated exposure of the CS (e.g., tone) in the absence of the US (e.g., shock). The ILc has been implicated in the recall of extinction memory and in inhibition of the original fear response (for a comprehensive review, see Milad and Quirk, 2012). Consistent with this, activity in the human subgenual (sg) ACC (sometimes referred to as vmPFC^1^), the putative homolog of ILc (Milad and Quirk, 2012) has been shown to correlate positively with extinction recall (it should be noted though that Myers-Schulz and Koenigs, 2012, suggest that, based on imaging data, the human homolog of ILc lies anterior to area 25 and includes caudal area 14). Conversely, PLc has been implicated in the maintenance of conditioned fear and the disruption of fear extinction in rodents (Burgos-Robles et al., 2009; Milad et al., 2014). However, contrary to cytoarchitectonic classifications (Vogt and Paxinos, 2014) functional neuroimaging studies implicate human midcingulate cortex (anterior division according to Vogt and Paxinos, 2014) as the likely functional homolog to PLc (Milad and Quirk, 2012). Both ILc and PLc send projections to inhibitory GABAergic neurons within the amygdala, as well as direct projections to the hypothalamus and brainstem, and it is these projections that appear to underlie the critical functional role of these vmPFC brain regions in the consolidation, retention, and expression of fear extinction (Milad et al., 2006).

The second major source of understanding of PFC and ACC regulation of negative emotion has come from human functional neuroimaging studies that have focused on the cognitive control processes regulating negative emotions including attentional deployment, re-appraisal and response suppression. These have implicated not only vmPFC, but also vlPFC and OFC as well as dlPFC (for comprehensive review, see Ochsner et al., 2012). Such findings are consistent with the structural and functional changes identified within ventral PFC in patients with mood and anxiety disorders (for review, see Milad and Rauch, 2007). The few, but insightful reports of impairment in emotional processing in patients with prefrontal damage support the hypothesis that the ventral PFC is an important component of the neural circuitry regulating emotions. For instance, it has been reported that distinct prefrontal regions are involved in dissociable processes of facial emotional expressions (Tsuchida and Fellows, 2012). When asked to detect the emotion expressed on a face and to discriminate between the specific emotions expressed, patients with lesion of OFC (areas 11, 13 and 14) and/or vmPFC (area 25 and subcallosal portions of areas 24 and 32) were impaired in detecting subtle emotions. On the other hand, patients with sustained damage in left vlPFC (areas 44, 45, 46 and 46/9, and part of anterior insular cortex) were able to detect the presence of emotional signals but had difficulty discriminating between specific emotions. Moreover, patients with damage in vmPFC, extending into the lateral orbital surface (area 11 and 13) and frontal pole (area 10) were impaired in directing visual attention during facial emotion identification, especially to a fearful face (Wolf et al., 2014). These findings are consistent with earlier studies demonstrating that patients with bilateral OFC lesions (areas 10, 11, 12 and 25) were impaired in emotional voice discrimination, identification of emotional facial expression, social behavior, and the subjective experience of emotion, when compared with the performances of patients with other prefrontal damage including dlPFC and mPFC (Hornak et al., 2003). Patients with OFC lesions (areas 10, 11, 12, 13 and 25) have also reported significantly greater subjective emotional experiences of anger than non-OFC damaged patients and significantly less feeling of happiness than healthy controls (Berlin et al., 2004). Of particular relevance to our discussion below of studies of fear and anxiety in animals, a more recent investigation of brain damaged subjects, has implicated vmPFC/lateral OFC in coordinating the neural and physiological responses to ambiguous cues (Motzkin et al., 2014). Whilst such studies implicate the ventral PFC in the control and regulation of emotion, these lesions are caused by traumatic events such as accidents and illnesses, resulting in widespread damage across multiple brain regions including damage to the underlying white matter. This highlights the need for experimental studies in animals that allow us to examine the effect of circumscribed prefrontal lesions using precise laboratory techniques to delineate functional differentiations between the specific regions more accurately.

However, the role of regions of PFC outside of the vmPFC, including OFC and vlPFC, remain relatively neglected in studies of negative emotion regulation in rodents, and whilst their role in higher order cognitive function and reward processing have been studied extensively in non-human primates, their contribution to negative emotion has received little attention. In this review, we first bring together the extant data from studies of ventral PFC damage in macaques and rats. We then compare them to more recent findings from our laboratory in marmoset monkeys.

## Orbitofrontal Cortex involvement in Fear and Anxiety: insights from Old World Monkeys

Negative emotional responses in macaques have been measured, traditionally, in response to an unknown human intruder or a model snake (Figure 1, upper panel). In a typical human intruder test (HIT), an animal is placed in a novel cage in a test room and their behavioral responses recorded (i) prior to the entry of an unfamiliar human; (ii) in the presence of the unfamiliar human during which they show his/her profile toward the animal (no eye contact); and (iii) the human staring at the animal. Increased duration of freezing and cortisol and reduced frequency of cooing vocalization in the “no eye contact” condition are associated with a high anxious temperament in rhesus monkeys (Kalin and Shelton, 1989; Kalin, 1993; Kalin et al., 1998; Shackman et al., 2013). However, studies of the effect of damage to ventral PFC on this anxiety test have been inconclusive (Figure 1, upper panel). Large aspirative lesions including orbital parts of area 47/12 and areas 11, 13 and 14 lead to reduced freezing in the presence of the human intruder (Kalin et al., 2007; Machado and Bachevalier, 2008), an effect replicated with more circumscribed aspirations of area 11 and 13, with variable and limited damage to adjacent 47/12, 14 and agranular insula (Machado and Bachevalier, 2008). Reduced freezing was not seen however, following aspirations restricted to areas 11, 13, 14 and caudal 10 (Izquierdo et al., 2005); although the latter did report increased mild aggression, relative to controls. Similarly, food retrieval latencies did not differ significantly in the presence of an unknown human, in comparison to controls, following aspirations of 11, 13 and 47/12 (Rudebeck et al., 2006). These differences are not easily explained by varying extents of damage. However, overall sensitivity to a human intruder can be highly variable between animals (see the section below “Orbitofrontal and ventrolateral prefrontal contributions to the regulation of conditioned fear, innate fear and anxiety”) and with the relatively low n’s in primate studies, such variability could contribute to null results, especially in those cases when the test was only administered post-surgery, so the animal’s “baseline” response couldn’t be taken into account.

More consistent effects of ventral PFC damage have been reported on the defensive response to the presentation of a snake, an innate fear stimulus (Figure 1, upper panel). Typically, an animal is tested in the Wisconsin General Test Apparatus (WGTA) and latencies to reach for a food item with or without the presence of a snake (usually a model snake) are compared. Studies with large aspirative lesions of areas 11, 47/12, 13 and 14, reported a reduction in snake fear, i.e., lesioned animals were quicker to retrieve food items in the presence of a snake than unoperated controls (Kalin et al., 2007). Similar results of blunting of fear have been reported after aspirations of areas 11, 47/12 and 13 (Rudebeck et al., 2006), and areas 11, 13, 14 and caudal 10 (Izquierdo et al., 2005). More circumscribed aspirations of areas 11 and 13, on the other hand, did not produce a blunting of the fear response (Machado et al., 2009) and neither did aspirations restricted to area 14 (Noonan et al., 2010). At first glance these results might indicate that either extensive damage to the entire ventral region or alternatively damage to area 47/12 specifically, may underlie the observed effects. However, all of the results described so far for both HIT and snake tests (except Machado et al., 2009) involved aspirative lesions that not only destroy local tissue but also damage white matter pathways running through the area connecting the temporal and frontal regions. Thus, it cannot be ruled out that the observed deficits were due to extraneous disconnections of neighboring regions. Indeed, when fiber-sparing excitotoxins, rather than aspiration, were used to destroy cell bodies within areas 11, 13 and 14, the blunted fear response to a model snake that had been seen following aspiration was not replicated; although blunting was seen if, in an attempt to mimic the damage caused to fibers of passage by the original aspirative lesion, a narrow strip of tissue in posterior OFC was aspirated (Rudebeck et al., 2013).

In summary, the blunting of the fear response is most likely the result of damage to fibers passing to and from the PFC causing a generalized reduction in arousal. This is particularly likely since much of the monoaminergic innervation of the PFC passes through these posteroventral regions.

## Orbitofrontal Cortex involvement in Fear and Anxiety: insights from Rodents

Like primates, rodents have a ventral PFC composed of a number of discrete cytoarchitectonic regions (see Figure 1, lower panel) but how these regions compare to those of primates remain unclear. There are two main schools of thought. Since rodent OFC is entirely agranular, one proposal is that the discrete regions are likely to be most similar to those agranular regions located in caudal aspects of primate OFC (Wise, 2008). A slightly revised hypothesis suggests that, based on connectivity patterns, medial orbital area (MO) and ventral orbital area (VO) in rodents may be similar to regions within primate area 14, rodent ventrolateral orbital area (VLO) and lateral orbital area (LO) to regions within primate area 13, and rodent dorsolateral orbital area (DLO) to the orbital sector of primate area 47/12 (Price, 2007). However, the extent of that similarity is an ongoing question since these regions may have evolved differently across species, with the expansion of PFC in primates allowing for the separation and development of cognitive functions that are more closely integrated in rodents. Indeed, such a hypothesis has also been proposed to explain differences that may arise between the prefrontal cognitive functions of non-human primates and humans (Teffer and Semendeferi, 2012).

It is difficult to make direct comparisons between the effects of manipulations of rodent OFC on negative emotion with those of primate OFC, not least because the tests used and responses measured to study negative emotion across these species vary considerably. Avoidance of mild, potentially threatening, unconditioned stimuli or contexts, as tested with the elevated plus maze and open field is unaffected in OFC lesioned rats with excitotoxic lesions targeting either LO and AI/DLO (Lacroix et al., 2000) or primarily LO and VO (Rudebeck et al., 2007; Orsini et al., 2015); although the latter (Rudebeck et al., 2007) did reduce the time taken to begin eating in a mildly anxiogenic environment (Figure 1, lower panel). These anxiety tests are most comparable to the HIT of anxiety in macaques, although the anxiety-like responses elicited by the human intruder are probably learned/conditioned, based on previous experience with humans, rather than largely unconditioned, in the case of the elevated plus maze and open field. In contrast, lesions of AI/DLO in rodent OFC, have been reported to heighten conditioned freezing responses to both contexts and cues (Lacroix et al., 2000) in simple Pavlovian conditioning, and lesions largely restricted to LO heighten and cause generalized freezing in Pavlovian discriminative contextual conditioning (Zelinski et al., 2010). In contrast, no effects were seen on acquisition of freezing to a Pavlovian conditioned cue following electrolytic lesions of LO/AI/DLO (Morgan and LeDoux, 1999; Tian et al., 2011). However, in mice, excitotoxic lesions of LO impaired the re-evaluation of a conditioned fear context (Costanzi et al., 2014). Such mixed results have also been seen with respect to MO. Permanent electrolytic lesions of this region had no effect on either acquisition or the next day expression of conditioned freezing to a Pavlovian cue (Sierra et al., 2015) whilst temporary inactivation of MO reduced conditioned freezing on the next day expression of a previously acquired conditioned freezing response (Rodriguez-Romaguera et al., 2015). This variability within rodent studies may be due in part to differences between the precise lesion location, although in many cases the lesions overlap considerably (e.g., Lacroix et al., 2000; Tian et al., 2011). Alternatively, differences in the test procedure, including whether conditioning was contextual or cued, or the overall strength of conditioned freezing may account for the discrepancies. For example, freezing levels were higher in control animals in those studies that found no effect of LO/AI/DLO lesions on single cue Pavlovian conditioning (Morgan and LeDoux, 1999; Tian et al., 2011) compared to the study that showed heightened freezing (Lacroix et al., 2000) and thus heightened freezing may not have been seen in the former because of ceiling effects. Similarly, only one study has investigated discriminative contextual fear conditioning and showed that despite similar overall levels of freezing to controls, freezing in the lesioned animals had generalized to the CS- (Zelinski et al., 2010), an effect that could not be seen in Pavlovian conditioning with a single cue or context.

In summary, the data reviewed so far does not produce a parsimonious account of the role(s) played by ventral PFC in the generation and regulation of fear- and anxiety-like responses either between or within different species. Reductions in innate fear appear to be the most consistent effect of aspirative lesions of the OFC in macaques, (although such effects have not been replicated with excitotoxic lesions) whilst in rodents, if anything, OFC lesions lead to heightened or generalized conditioned fear responses, alongside intact unconditioned anxiety responses. One obvious distinction between species is that conditioned fear responses have been the focus of rodent studies, whilst innate fear and experience-based (learned) anxiety-like responses have been more commonly used in macaques, components of negative emotion which may well involve distinct, although overlapping circuitry (see Davis et al., 2010). Consequently, we have developed a program of work to bridge the gap between rodent and primate studies by developing a battery of tests of negative emotion that include innate fear, experience-based anxiety and conditioned fear in a new world primate, the common marmoset. Core to emotion, and its regulation, is control of peripheral arousal with major re-entrant feedback loops between brain and body underlying emotional states (Salzman and Fusi, 2010). Dysfunction of this feedback contributes to emotion dysregulation (Makovac et al., 2015), but physiological indices of emotion were rarely measured in the studies of orbitofrontal and ventral PFC emotion regulation in animals described above, despite being the primary measure of negative emotion in humans. An exception was Machado and Bachevalier (2008) which showed reduced blood cortisol reactivity during social isolation following OFC lesion in rhesus monkeys. Thus, in order to improve translation from experimental animal studies into the clinic, we include both physiological as well as behavioral measures of the emotional response.

## Orbitofrontal and Ventrolateral Prefrontal Contributions to the Regulation of Conditioned Fear, innate Fear and Anxiety

**Figure 2:**
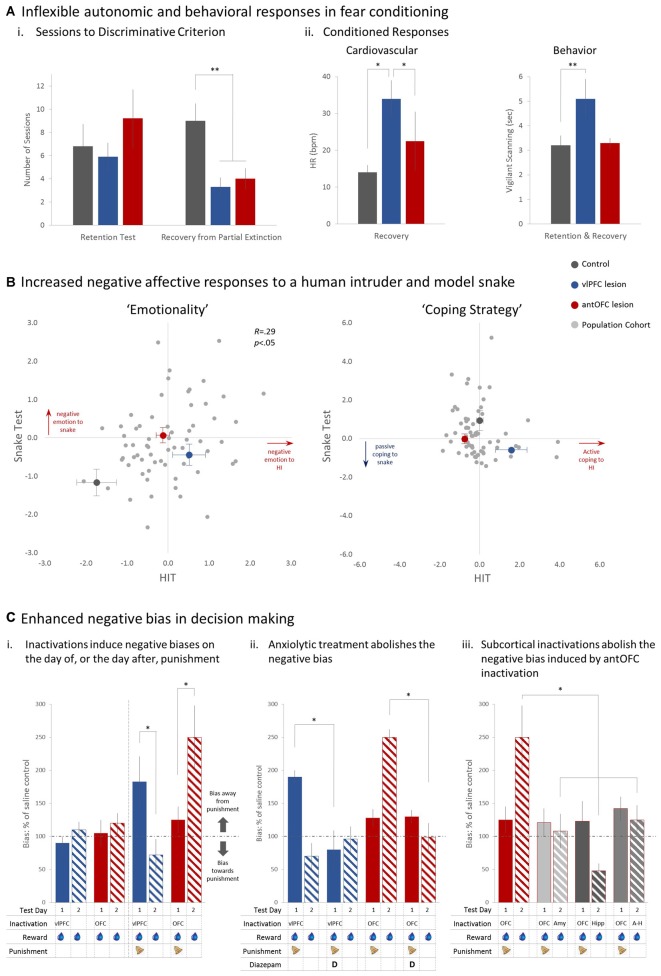
**The effects of permanent and temporary manipulations of the antOFC and vlPFC on the responsivity of marmosets to a variety of fear and anxiety-inducing stimuli.** In **(A)** both lesioned groups, post-surgery, took the same number of sessions as controls to regain discriminative conditioned responding to a CS associated with aversive loud noise (**i** “Retention”). Following exposure to one session of partial extinction, however, whereas controls adapted their responding and took many sessions to then regain their discriminative responding (**i** “Recovery”), the lesioned groups maintained strong discriminative conditioning throughout **(ii)**. *p < 0.05, **p < 0.01. Figures redrawn from Agustín-Pavón et al. (2012). In **(B)** pale gray dots represent the emotionality and coping strategy component scores of individual marmosets in the colony in response to a human intruder (HIT) and a model snake. Emotionality scores show a significant positive relationship such that animals scoring high on the HIT, score high on the Snake too and *vice versa*. Superimposed on these scores are the average scores of each of the lesioned and control groups. Both lesioned groups showed greater emotionality scores on the HIT and snake test whilst their coping strategies differed across the two tests. The vlPFC lesioned group displayed a higher, more active coping strategy on the HIT but both antOFC and vlPFC lesioned groups displayed a lower, more passive strategy score on the Snake, compared to controls. Data taken and redrawn from Agustín-Pavón et al. (2012) and Shiba et al. (2015). In **(C)** the effect of temporary inactivation of the antOFC and vlPFC on the effects of cost-benefit decision making are displayed. Inactivation of the vlPFC had no effect on response bias during reward only sessions (**i**, left side) but in the presence of punishment (Test Day 1) significantly increased responding away from punishment (**i**, right side). antOFC inactivations also had no effect on response bias on reward only sessions but enhanced responding away from the punished side the day after having received punishment (Test Day 2). The biases in both cases were ameliorated with concomitant treatment with the anxiolytic, diazepam **(ii)**. The antOFC-induced punishment bias on the day after punishment was blocked by inactivation on Test Day 2 of the amygdala (Amyg) bilaterally, anterior hippocampus (Hipp) bilaterally, or amygdala and hippocampus unilaterally on opposite sides of the hemisphere (A-H disconnection; **iii**). The key below each graph in **(C)** indicates when and where infusions were made and whether punishment was present or not. *p < 0.05 on square-root transformed data. Figures redrawn from Clarke et al. (2015).

When presented with two auditory cues, one of which is associated with an aversive loud noise (US+) marmosets develop discriminative conditioned heart rate (HR) and vigilant scanning responses to the auditory cue associated with the US+ (CS+; Mikheenko et al., 2010; Agustín-Pavón et al., 2012). These Pavlovian conditioned responses (CRs) remain unchanged in the retention test following localized excitotoxic lesions of either the anterior OFC (area 11) or vlPFC (area 47/12; Figure 2Ai, “Retention”). To create a condition of increased uncertainty, the animals were subsequently exposed to a session of partial extinction in which CS+ and US+ were partially decoupled, with two of the four CS+s no longer leading to aversive noise. This reduced the impact of the CS+ as a danger signal, and consequently, the control group required many additional sessions of conditioning to regain their discriminative CRs. This was not the case in the lesioned groups, which maintained a relatively rigid conditioned discriminative fear response in subsequent sessions despite experiencing the altered contingencies (Figure 2Ai, “Recovery”). Moreover, the continued exposure to the Pavlovian fear-conditioning paradigm resulted in those animals with lesions of the vlPFC displaying heightened HR and vigilant scanning responses (Figure 2Aii). These effects appear similar to those described in rodents where heightened conditioned freezing responses were observed following OFC excitotoxic lesions (Lacroix et al., 2000), but are inconsistent with the reports of blunted innate fear and anxiety responses in macaques with OFC damage (Izquierdo et al., 2005; Rudebeck et al., 2006; Kalin et al., 2007).

To address this discrepancy, innate fear and anxiety was also measured in the same groups of animals. Marmosets display a rich and varied repertoire of behaviors to snakes (innate fear: Shiba et al., 2015) and human intruders (anxiety: Agustín-Pavón et al., 2012; Mikheenko et al., 2015), which can be distilled by a principle component analysis into two major components reflecting “emotionality” and “coping strategy” (Shiba et al., 2014). Maintaining a large distance from the fear- or anxiety-inducing stimulus, along with reduced locomotion, increased attention (head bobbing in HIT; head cocking in Snake Test) and vocalizations (egg calls in HIT; tsik-egg calls in Snake Test), typify high scores on the emotionality component. In contrast, high scores on the strategy component reflect primarily the number of mobbing calls made (tsik or tsik-egg) which act to alert other marmosets and drive the intruder/snake away. There are large individual differences in the level of emotionality displayed by marmosets (see gray circles in Figure 2B, “Emotionality”) with a significant positive correlation between emotionality scores on the HIT and snake test. Similar scores across repeated testing suggest that these scores may reflect an emotionality/anxiety trait (Shiba et al., 2014; Mikheenko et al., 2015), similar to that already described in rhesus monkeys (Kalin and Shelton, 1989; Shackman et al., 2013) and which, in humans, has been shown to be associated with over-generalization of fear responses and to act as a vulnerability factor for developing mood and anxiety disorders (Chambers et al., 2004; Sandi and Richter-Levin, 2009). Indeed, we have shown that those marmosets showing high levels of emotionality to the snake show fear generalization on the Pavlovian discrimination task (Shiba et al., 2014) similar to that reported in rats with a high anxiety phenotype (Duvarci et al., 2009) and individuals with anxiety disorders including panic disorder (Grillon et al., 2008; Lissek and Grillon, 2010), PTSD (Grillon and Morgan, 1999; Jovanovic et al., 2010; Mauchnik et al., 2010), and GAD (Lissek et al., 2014).

When testing the anterior OFC (antOFC) lesioned and vlPFC lesioned groups on these fear- and anxiety-inducing tests, both lesioned groups displayed heightened emotionality to both the human intruder and the snake (see larger colored circles in Figure 2B, “Emotionality”) but whilst vlPFC lesioned animals displayed increased tsik-egg vocalizations and thus an active (higher) coping strategy score on the HIT, both groups showed a significant reduction in tsik calls in the presence of the snake, resulting in a more passive (lower) strategy score (Figure 2B, “Coping Strategy”). Together, these findings implicate both regions of ventral PFC in the generation/regulation of negative emotional responses and reveal that reduced activity within these regions can lead to heightened anxiety and more rigid fear responses reminiscent of the pattern of deficits seen in patients with emotional disturbance, including those with mood and anxiety disorders. By comparing the levels of lesion-induced emotionality with the “normal” range of emotionality in a large cohort of monkeys within the colony (Figure 2B), it can be seen that the levels obtained in animals with lesions of ventral PFC remained within the “normal” range, tending towards levels of emotionality seen in intact individuals considered to have high trait emotionality. These findings are consistent with a recent report of reduced functional activity in ventral PFC in humans with high trait anxiety (Indovina et al., 2011) but extend this result by suggesting that damage to two distinct regions of ventral PFC can lead to a similar high emotionality/anxiety phenotype. They also implicate both these regions in the selection of coping strategies to potential threat (for detailed discussion, see Shiba et al., 2015).

The question arises as to why these findings of heightened fear and anxiety have not so far been reported in previous studies of OFC damage in macaques? We suggest three main reasons. First, the overall blunting of emotional responses that have been reported after large aspirations of the OFC in macaques that damage multiple cytoarchitectonic areas (see Figure 2, upper panel) are most likely the result of a reduction in arousal, as a consequence of the disruption of fibers passing through to other regions of the PFC, effectively causing a partial de-afferentation and de-efferentation. Second, the lack of effects on negative emotion with more selective excitotoxic lesions of lateral OFC (areas 11 and 13) may be a consequence of large individual variability in emotional responses, making it difficult to detect heightened responses in “between” group studies with small sample sizes. Alternatively, such lack of effects may be a consequence of opposing contributions of area 11 and 13 to the regulation of negative emotion, acting to cancel each other out? Unfortunately, excitotoxic lesions restricted to area 11, as in the marmoset, have not yet been studied in the context of negative emotion in macaques.

## Differential Contributions of the Orbitofrontal and Ventrolateral Prefrontal Cortex to Approach-Avoidance Decision-Making

Whilst lesions of antOFC and vlPFC induce what appears to be a very similar pattern of deficits in negative emotion, as measured by tests of discriminative conditioned fear, innate fear and anxiety, the question remains as to the unique contribution played by the antOFC and vlPFC in regulating emotionality. We have recently addressed this issue by comparing the effects of inactivating these two regions independently on an approach-avoidance, cost-benefit, decision-making task (Clarke et al., 2015). Heightened anxiety has been shown to bias decision-making away from punishment (Mitte, 2007) and thus it would be predicted that inactivation of either region would induce a punishment bias by virtue of the increase in anxiety. Animals were trained to respond to two identical stimuli for reward on a variable interval schedule, whereby the first response after a variable time interval had elapsed resulted in banana milkshake. As the reward schedules were independent of each other the optimal strategy for a marmoset was to respond to both stimuli to maximize reward availability. Occasional probe sessions were presented in which, superimposed over the reward schedule was a punishment schedule, whereby an aversive loud noise was associated with responding to one of the stimuli, but not the other, affording the animal the opportunity to avoid the noise. Whilst inactivations of either the antOFC or vlPFC with a GABA A/B agonist mix had no effect on responding for reward *per se*, they did affect the responsivity of the animals to punishment. Inactivation of either region enhanced avoidance of the aversive noise resulting in a bias of responding away from the punished side (Figure 2Ci) that was ameliorated with the anxiolytic, diazepam (Figure 2Cii), and thus consistent with the hypothesis that enhanced anxiety can induce a negative or punishment bias. However, whereas the negative bias following inactivation of vlPFC was apparent on the day of punishment, it was only seen on the day after punishment following antOFC inactivation. Moreover, this next day bias away from the punished side induced by antOFC inactivation was dependent for its expression upon a circuit involving the amygdala and anterior hippocampus. Bilateral inactivation of either the amygdala or anterior hippocampus or disconnection of the two, using a crossed unilateral inactivation procedure, on the day after punishment abolished the “next day” punishment bias induced by antOFC inactivation (Figure 2Ciii). Thus, taking vlPFC off-line had a direct effect on an animal’s decision-making abilities to choose between reward and punishment. In contrast, taking antOFC off-line did not alter decision-making at the time of the inactivation, but affected the memory of the punishing experience, which impacted upon “next day” behavior and depended upon an interaction between the anterior hippocampus and amygdala for its expression.

## Distinct Cognitive Deficits Underlie increases in Negative Bias induced by inactivation of Orbitofrontal and Ventrolateral Prefrontal Cortex

Only a few neural intervention studies have specifically targeted the vlPFC of primates (e.g., Iversen and Mishkin, 1970; Jones and Mishkin, 1972; Kowalska et al., 1991; Rushworth et al., 1997; Buckley et al., 2009; Baxter et al., 2009). Of particular relevance to the present discussion is the disruption of attention towards higher-order relevant reward cues in the environment caused by excitotoxic lesions of the vlPFC in marmosets (Dias et al., 1996, 1997; Wallis et al., 2001) and the failure to shift attention away from salient features of the environment. Since aversive stimuli are salient features of any environment and naturally attract an animal’s attention, then an impairment in shifting attention away from the salient punishment in the decision-making task described here could account for the enhanced punishment bias induced by vlPFC inactivation. Continued attention towards the punishing stimulus at the expense of the reward would not only impair the cost-benefit analysis by allowing the subject’s choice to be unduly influenced by the punishment but would also lead to heightened anxiety. Such a hypothesis is consistent with the activation of this region in human cognitive re-appraisal studies (Buhle et al., 2013) in which subjects have to shift their attention to potentially less salient but more positive aspects of a negative picture in order to diminish its negative affect. Indeed, increased activation of the vlPFC when using cognitive re-appraisal to suppress negative emotion whilst viewing stressful and upsetting pictures was associated with a greater reduction of self-reported stress in trait anxious individuals (Campbell-Sills et al., 2011). This may seem at odds though with the finding of vlPFC hyperactivity in response to emotionally laden stimuli in a number of studies of patients suffering from anxiety disorders (Dilger et al., 2003; Koric et al., 2012) and individuals with high trait anxiety (Telzer et al., 2008). However, rather than the hyperactivity being a direct neural correlate of increased anxiety it is more likely a compensatory mechanism. For example, patients with GAD that showed the greatest vlPFC activation in response to the presentation of angry faces and exhibited greater attention bias away from the angry faces had less severe symptoms (Monk et al., 2006). Also, in GAD patients, effective mindfulness-based intervention is found to be associated with increased vlPFC activation and enhanced PFC-amygdala functional connectivity (Hölzel et al., 2013). Thus, we suggest that the vlPFC contributes to the regulation of emotion as a consequence of its involvement in higher-order attentional control (Clarke et al., 2015).

It is interesting to note that a similar role in attentional control has recently been proposed for the PLc in the regulation of fear conditioning in rodents (Sharpe and Killcross, 2015a,b). Rat and mouse PL/ILc has been implicated in shifting higher-order attentional sets (Birrell and Brown, 2000; Bissonette et al., 2008), a function likened to that associated with vlPFC in marmosets (Dias et al., 1996) and humans (Hampshire and Owen, 2006). However, comparing prefrontal regions across species based on similarity of functional deficits induced by localized lesions can be misleading. For example, besides being compared with vlPFC because of the similarity of set-shifting impairments, rodent PLc has been likened to human dorsal ACC (dACC), BA 24, based on their apparently similar contribution to the regulation of conditioned fear (Milad and Quirk, 2012), and the anterior part of human vmPFC (encroaching on BA 10/14; Balleine and O’Doherty, 2010) based on studies of contingency learning (Tanaka et al., 2008; Liljeholm et al., 2011). In contrast, consideration of cytoarchitecture and receptor distribution points to primate perigenual ACC (pgACC), BA 32, as equivalent to PL (Gabbott et al., 2003; Vogt et al., 2013), but the effects of lesions of primate pgACC have so far not been studied in the context of attentional set-shifting. Thus, the relationship between primate vlPFC and rodent PLc in relation to attentional control remains to be determined. However, vlPFC in primates does send projections to the medial PFC (Roberts et al., 2007) including the putative homolog of PLc (area 32; Vogt and Paxinos, 2014) and recent findings have implicated this perigenual region in primates in negative biases in decision-making (Amemori and Graybiel, 2012). Hence, it will be important in future studies to determine if and how these regions interact.

In contrast to the attentional control functions of the vlPFC, we suggest that the antOFC contributes to the regulation of negative emotion by virtue of its involvement in predicting future events. There have been a number of excellent reviews on the role of the OFC in object-outcome and outcome-value updating (Rudebeck and Murray, 2014), in representing reward relevant states (Schoenbaum et al., 2011) and in creating cognitive maps (Wilson et al., 2014). Common to all of these hypotheses is that the OFC is important in integrating biologically relevant information such as somatosensory and visual perception, past experiences and prediction error signal, and formulating the current state of the surroundings and the task at hand. Based on this current status, the OFC simulates potential future outcomes to guide decision-making. When placed in an uncertain situation involving potential threats, as in the approach-avoidance, decision-making task described here, cognitive abilities such as being able to learn and distinguish safety cues from danger signals in the environment, appropriate and accurate estimation of threat occurrences, and effective and efficient approach to and avoidance of probable threat, all contribute to establish an accurate prediction of future events, thus reducing uncertainty (Grupe and Nitschke, 2013). Inactivation of the antOFC is likely to have prevented the formation of such knowledge, increasing uncertainty and leading to increased anxiety and over-estimation of the negative outcome during the re-exposure to the same context the following day. Thus, a dysregulated antOFC may well underlie the over-expectation of negative outcomes not only in individuals with high trait anxiety (Mitte, 2007) but also in clinically anxious individuals (Warda and Bryant, 1998; Borkovec et al., 1999; Gilboa-Schechtman et al., 2000). Reflecting these findings, it has been suggested that high anxiety is associated with abnormality in the neural circuitry involved in the expected value calculation of future aversive events (Grupe and Nitschke, 2013). Evidence that such anxiety responses may be the result of unregulated bottom-up processing by subcortical circuits is the observation that inactivation of either the anterior hippocampus, amygdala, or disconnecting the two, blocked the behavioral avoidance induced by the antOFC inactivation (Clarke et al., 2015). Such unregulated bottom-up influences from these limbic structures biases the cognitive processing in an overly conservative fashion (Bishop, 2007; Grupe and Nitschke, 2013), including fear generalization, over estimation of threat cost and excessive avoidance. Instead of reducing uncertainty, these maladaptive responses result in maintenance of high uncertainty in which individuals are kept on high alert and under chronic stress. Being constantly overloaded physiologically and cognitively, these conditions can eventually lead to the development of affective disorders in these individuals (Sandi and Richter-Levin, 2009).

## Conclusion

Taken together, the effects of impaired functioning in the antOFC and vlPFC on conditioned and innate fear, anxiety and cost-benefit decision-making in the marmoset provide important new insights into the specialized roles played by distinct sectors of the ventral PFC in the regulation of negative emotion. First, they demonstrate a consistent heightening of sensitivity to conditioned fear and anxiety-provoking stimuli across a wide range of contexts and a variety of behavioral and autonomic measures bridging the gap between human and rodent studies and promoting both forward translation into the clinic and backward translation to the rodent. Second, they reveal the causal role of prefrontal regions beyond that of medial PFC in the regulation of negative emotion. Third, they highlight how a similar high anxiety phenotype may be caused by dysregulation within distinct sectors of PFC, which are the result of different underlying cognitive deficits. The latter has major implications for the successful treatment of disorders involving emotion regulation. One form of treatment, Cognitive Behavioral therapy (CBT) already recognizes the different aspects of a psychological disorder in terms of dysfunctional emotions, maladaptive behaviors and cognitive processes. By understanding how they are parsed within associative neural systems will bring us one step closer to individualized treatment strategies. For example, cognitive re-appraisal, requiring a patient to shift attention from negative to positive aspects of an emotional situation may be more successful in a patient poor at predicting (e.g., as a consequence of dysregulation in antOFC) than one deficient in attentional control (e.g., as a consequence of dysregulation in vlPFC). Thus, if we can fractionate the component cognitive processes that constitute the “top-down” regulatory mechanisms within ventral, dorsal and medial PFC, we will provide the necessary new insights into the varied causes of anxiety, which will allow for more precise stratification of the disorder, leading to individualized treatment strategies.

## Author Contributions

YS and ACR: manuscript writing and idea building. AMS: conceptual discussion.

## Conflict of Interest Statement

The authors declare that the research was conducted in the absence of any commercial or financial relationships that could be construed as a potential conflict of interest.

## Footnotes

^1^vmPFC usually refers to an area that includes the sgACC but extends anterior of the genu of the corpus callosum.
